# Bio-hybrid photoelectrochemical catalysis for solar fuels and chemicals conversion

**DOI:** 10.1038/s41467-025-64931-9

**Published:** 2025-10-14

**Authors:** Bin Cai, Mariia V. Pavliuk, Gustav Berggren, Haining Tian

**Affiliations:** 1 Department of Chemistry-Ångström Laboratory, Uppsala University, Uppsala, Sweden; 2 Institute for Energy Research, School of Materials Science and Engineering, Jiangsu University, Zhenjiang, Jiangsu China

**Keywords:** Bioinspired materials, Carbon capture and storage, Photobiology, Biocatalysis

## Abstract

Bio-hybrid photoelectrochemical (PEC) devices integrate the complementary advantages of both biocatalyst and abiotic components, providing opportunities for efficient catalysis under mild conditions with high selectivity and low over-potential. However, the practical applications of such devices depend on the stability and efficiency of the bio-abiotic interface, where suboptimal charge transfer, biocatalyst fragility, and scalability challenges persist. In this Perspective, we evaluate established strategies for wiring biocatalysts to electrode substrates within bio-hybrid PEC architectures, analyze their catalytic performance, and operational limitations, and underly mechanistic principles. Then, we highlight the integration of whole-cell biocatalysts with high-performance semiconductor scaffolds as a promising design paradigm, offering a scalable platform for sustainable, solar-driven chemical production.

## Abstract (web summary)

Bio-hybrid photoelectrochemical systems integrate microbial components with abiotic conductors/semiconductors for solar fuels and chemical conversion. Here, the authors analyze the bottlenecks related to catalytic efficiency, stability and scalability, and propose strategies to address these challenges.

## Introduction

The global energy crisis and growing carbon emissions have intensified the search for renewable energy solutions that can efficiently convert and store solar energy into chemical bonds. Among the various solar-driven technologies, photoelectrochemical (PEC) catalysis has emerged as a promising strategy for direct solar-to-chemical conversion, mimicking the natural process of photosynthesis^1–3^. Compared to homogenous photocatalysis, where product separation and catalyst recovery are often inefficient, PEC systems operate with catalysts immobilized on substrate, giving intrinsic spatial separation of oxidation and reduction reactions by reacting separately at working electrode and counter electrode. This architecture facilitates product separation, catalyst recovery, and suppresses unnecessary side reactions arising from product diffusion between electrode compartments^4^. Compared to photovoltaic-electrolyzer devices with same efficiency, PEC devices provide an integrated platform that unifies light absorption, charge separation, and catalytic reactions within a single architecture, potentially reducing system’s complexity and ground occupation associated with manufacturing of photovoltaic (PV) panels. However, conventional fully artificial or abiotic PEC systems still suffer from limited product selectivity, high overpotentials, and a dependence on critical metal catalysts. To address these limitations, bio-hybrid PEC systems have been regarded as an alternative approach, by combining the strengths of both natural and artificial components^5–11^. Such biohybrid systems integrate microbial components, spanning from monomeric and multimeric enzymes such as hydrogenase (H_2_ase)^12–16^, nitrogenase (N_2_ase)^17^, formate dehydrogenase (FDH)^18–21^ and photosystems (PSII)^22–24^, to subcellular organelles (thylakoids, chloroplasts, etc.)^25–27^, and even whole organisms (bacteria, algae, yeast etc.)^20,28,29^, with abiotic conductors/semiconductors. The aforementioned limitations of fully abiotic PEC systems can be overcome by employing biocatalysts, such as enzymes and whole- microorganisms, that enables high selectivity through precise control of reaction pathways^30^, and enhances energy efficiency by providing high catalytic rates at low driving force^31^. Moreover, biocatalysts suppress the need for critical metals, instead relying on metalloenzymes incorporating Fe, Ni, or Mn clusters as active sites for catalysis^32,33^.

Over the past decade, bio-hybrid PEC research has witnessed substantial progress, with emerging applications in reduction reactions, including hydrogen production^12–16^, CO_2_ reduction^18–21^, and N_2_ fixation^17^, etc. and in oxidation reactions, including oxygen evolution^22–24,26,28,34^, alcohol oxidation^35–37^, etc. Moreover, the device structures have gradually evolved from early single-component designs^12,13,15,34,35,38,39^ to bias-free tandem cells^14,16,18,20–23,40^ as well as scalable architectures capable of improved charge transport and catalytic efficiency^14,25,27,41,42^. These advancements have been promoted by the development of conductor/semiconductor materials, tailored substrate/biocatalyst interfaces, and biocatalyst with enhanced robustness and electron transfer properties. Despite these achievements, bio-hybrid PEC systems face several bottlenecks related to catalysis efficiency, stability, and scalability. Many of these limitations are critically related to the interface architecture and conditions. For example, through the implementation of proper interface strategies, photocurrents as high as 5 mA cm^−2^ have been achieved, alongside a solar-to-formate conversion efficiency approaching 1% in CO_2_ reduction bio-hybrid PEC systems^18^. By incorporation of cytochrome c (cyt c) as a redox mediator, bio-hybrid PEC system for quinone reduction with storage lifetime up to 2 years has been realized^43^. Additionally, scalable fabrication has been explored, exemplified by the construction of a 25 cm^2^ bio-hybrid PEC device interfaced with Os-complex polymers and deposited via sequential spray coating^42^. Collectively, these advancements reveal the critical role of efficient interfacial wiring in achieving high-performance, durable, and scalable bio-hybrid PEC architectures. In this Perspective, we review progress in bio-hybrid PEC systems during the last decade, with an emphasis on how the substrate/biocatalyst interfacial wiring strategies influence the above challenges and propose the most promising combination strategies for achieving high-efficiency bio-hybrid PEC catalysis.

### Bio-hybrid PCE systems

**Fig. 1:**
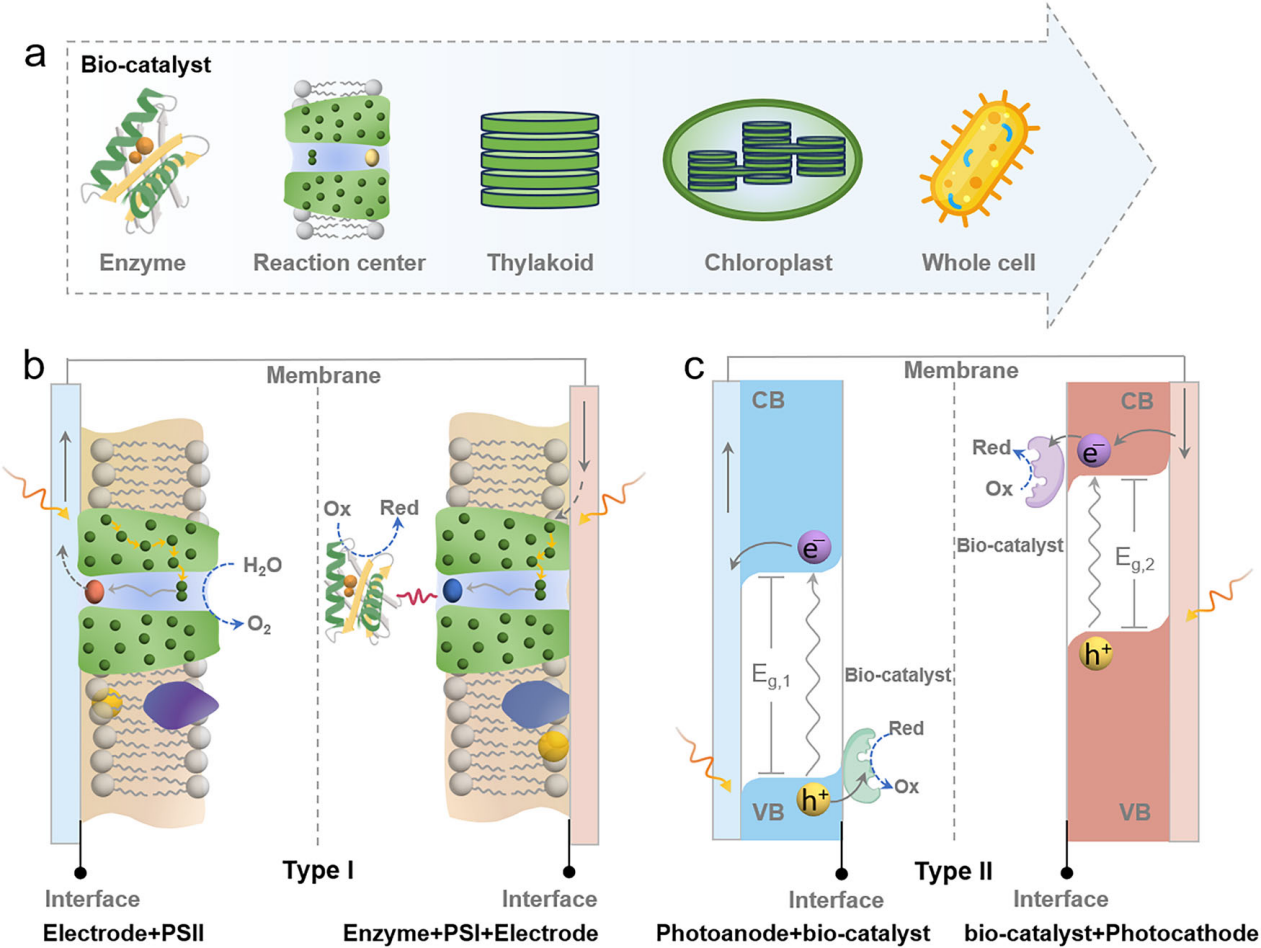
Composition of bio-hybrid PEC systems can be decoupled into bio-catalysts and light-harvesting substrates. **a** Representative biocatalyst schemes from subcellar to whole-cell system; Structure schemes of two kinds of bio-hybrid PEC systems: **b** Type I, abiotic conductive electrode + biotic catalyst system; **c** Type II, abiotic light-harvesting semiconductor + biotic catalyst.

Bio-hybrid PEC systems (Fig. 1) can be mainly divided into two categories: (i) abiotic conductive electrodes with an embedded biotic catalyst system (biotic light-harvesting center wired with enzymes, Type I)^19,24,28,35,39,44–47^; and (ii) abiotic light-harvesting semiconductors with biotic catalysts (Type II)^12–16,18,20,22,23,34,37,48,49^. In the former case, light harvesting and the catalytic reaction both happen in a biotic environment, and the abiotic substrate only acts as a conductive scaffold. PSI and PSII are the most widely applied light-harvesting and charge-separation centers in Type I assemblies, which can be further coupled with a variety of enzymes to perform desired PEC reactions. More specifically, the excited-state light harvester in PSI (P700*) shows a reduction potential around −1.2 V *vs*. standard hydrogen electrode (SHE), and an internal quantum efficiency (IQE) of almost 100%, thus acting as a powerful light-driven source of high reduction potential electrons^50,51^. Linking PSI with H_2_ase, FDH, or laccase can be used for photocatalytic production of hydrogen, formic acid, or hydro-peroxide, respectively. The excited state light harvester in PSII (P680*) has an oxidation potential of 1.2 V *vs*. SHE, which is used to drive the oxidation of the oxygen-evolving cluster, the only biocatalyst in nature that splits water^52^. While the IQE of PSI and PSII are extremely high, their overall solar-to-fuel conversion efficiencies are relatively low, with reported photocurrents in the range of nA∼μA cm^−2^, i.e., far below the standard for industrial applications.

To maintain overall functionality, natural photosynthetic systems have evolved into complex environments^53^. While certain components, such as accessory pigments, protein matrices, and metal ions, may not directly participate in the primary photochemical reactions or may even reduce light-harvesting efficiency, they play essential roles in thermal regulation, structural stability, and energy transfer^54,55^. The relatively low light-harvesting efficiency is an important factor that limits the performance in type I bio-hybrid PEC, due to the narrow spectral absorption (e.g., PSI and PSII have minimal absorption gap between 500–650 nm) and low light-intensity tolerance (< 10 mW cm⁻^2^). Type II bio-hybrid PEC systems overcome this limitation by applying an artificial light-harvesting semiconductor as the substrate, including photoanodes (e.g., BiVO_4_, In_2_S_3_, PbS, TiO_2_/dye, etc.)^22,23,34,37,48,56^ and photocathodes (e.g., Cu_2_O, p-Si, NiO/dye, etc.)^12,13,15,16,18,20^, coupled with enzymes or microorganisms. As such, type II devices represent an attempt to fully integrate the complementary advantages of both abiotic and biotic components. It utilizes the high selectivity, minimal driving force requirements, noble metal-free and mild operational conditions of biocatalysts; Simultaneously, it retains the superior light-harvesting efficiency and charge-separation capabilities of semiconductor materials, enabling enhanced solar-to-chemical energy conversion with high material design flexibility.

### Challenges in achieving efficient interfacial engineering

**Fig. 2:**
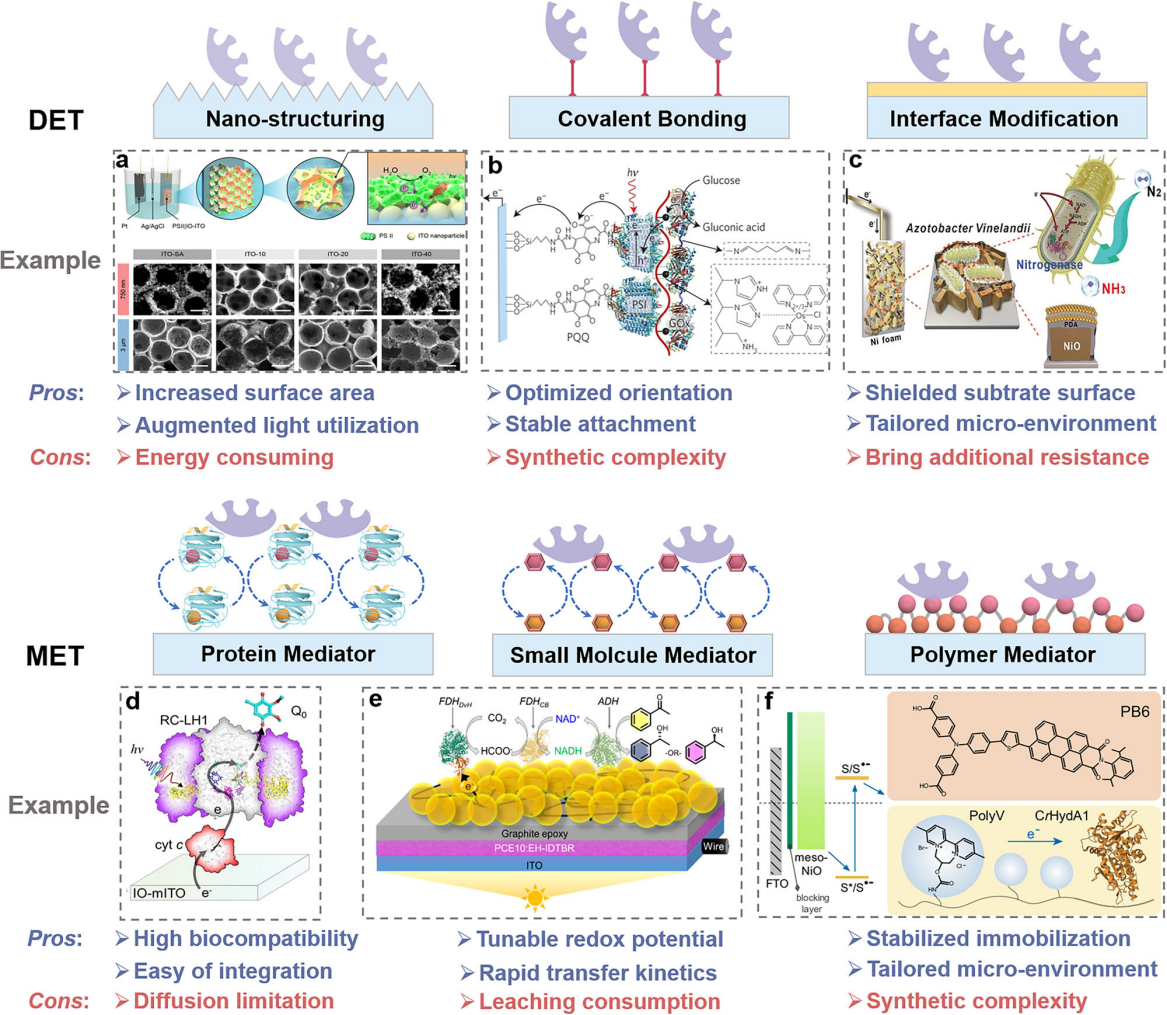
Strategies to wire the abiotic substrate and biocatalyst are critical to bio-hybrid PEC devices. Representative examples, advantages, and disadvantages of direct electron transfer (DET) strategies, including **a** Substrate nano-structuring, **b** Covalent bonding between the biocatalyst and substrate, **c** Interface modification with functional materials; and mediated electron transfer (MET) strategies, including **d** Protein mediator, **e** Small molecule mediator, **f** Polymer mediator. **a**, **e** reproduced from Fang et al.^24^ and Bouwens et al.^69^, respectively. **b**, **f** reproduced from Efrati et al.^35^ and Cheng et al.^16^, respectively; Copyright (2025) Springer Nature Ltd; and **c**, **d** reproduced from Zhang et al.^17^ and Friebe et al., respectively; Copyright (2025) Wiley-VCH.

Though bio-hybrid PEC system represents a highly promising solar energy conversion technique, several limitations related to efficiency, stability, and scalability need to be solved before the technology can move towards large-scale applications. Specifically, the relatively poor charge transfer kinetics at the abiotic substrate/biocatalyst interface often results in significant energy loss and interfacial charge accumulation, reducing overall efficiency and inducing photodamage. Moreover, desorption or deactivation of biocatalysts over time due to poor interfacial biocompatibility or unstable interactions can lead to decreased operational durability. Also, the normally used drop-casting deposition technique limits homogeneous biocatalyst loading over a large area. Developing strategies to finely wire abiotic substrates with biocatalysts is highly desirable, as it can enhance interfacial charge transfer, tailor the local microenvironment, and enable scalable fabrication of bio-hybrid devices. However, achieving such integration remains a significant challenge. Specific strategies to address the above challenges will be summarized in the next section (Fig. 2).

## Strategies for wiring abiotic-bio interface

### Direct electron transfer

Substrate nano-structuring: Surface nano-structuring offers an effective strategy to increase substrate surface area. In the DET mechanism, biocatalysts are expected to closely attach to the substrate, where a larger surface area can enhance biocatalyst loading amount, thereby improving performance^13,14,18,19,24,36,38,57,58^. For instance, Zhao et al.^13^ reported that when a Si substrate surface was modified into a nano-porous morphology via a Ag-assisted etching process, the resulting H_2_ase-functionalized Si photocathode generated a photocurrent exceeding 1 mA cm^−2^, surpassing previously reported systems by orders of magnitude. Conversely, they found a significant photocurrent loss after reducing the Si substrate’s surface area through additional chemical etching, suggesting the critical role of enlarging the substrate surface area for biocatalyst immobilization. Though nano-structuring through surface-etching is effective, its applicability is usually material-dependent. Alternative approaches involve depositing a layer of three-dimensional (3D) macro-porous scaffolds (e.g., TiO_2_^14,18,36^, ITO^19,24,57,58^, or carbon^46^) above the substrates. For example, Moore et al.^18^ introduced a perovskite photocathode, a highly efficient semiconductor developed during the last decade, coated with a macro-porous TiO_2_ layer to immobilize FDH enzymes, achieving a photocurrent of 5 mA cm^−2^ at 0.4 V *vs*. reversible hydrogen electrode (RHE). Remarkably, they achieved a solar-to-formate energy conversion efficiency of nearly 1% after integrating this FDH/TiO_2_/perovskite bio-hybrid photocathode with a BiVO_4_ photoanode into a bias-free tandem device. Further studies by the same group systematically explored the structure-activity relationship of 3D surface based-substrates, where they fabricated porous structures with varied size cavities above the substrate by tuning the combination of different size polystyrene beads and ITO nanoparticles^24^. Their findings revealed that cavity size directly affects biocatalyst loading and retention efficiency: after PEC tests, less than 1% of PSII was released from the 750 nm porous ITO electrode, while 22% desorbed into the electrolyte from the 3 μm porous ITO electrode. These results highlight the importance of tailored substrate surface morphology on the bio-PEC device performance. Nevertheless, given that most substrate nano-structing methods are energy consuming processes, developing fabrication approaches at low temperature will broaden their applicability and scalability.

Covalent-bond linking: The active sites of a biocatalyst are often structurally buried, requiring optimal orientation on the substrate to shorten the charge transfer distance for efficient DET^59^. Covalent bonding can control the orientation of biocatalyst through choice of binding site(s), ensuring that its active sites are properly aligned with the substrate. Additionally, the strong covalent interactions help to establish durable substrate-biocatalyst interfaces, potentially improving device stability^34,35^. However, unlike the non-directional nature of electrostatic interactions, covalent linking requires a carefully tailored chemical environment to ensure site-specific attachment. For instance, Tapia et al.^34^ reported the covalent immobilization of laccase on In_2_S_3_ semiconductor via 1-ethyl-3-(3-dimethylaminopropyl) carbodiimide hydrochloride (EDC) crosslinking chemistry and explored the laccase-based bio-hybrid PEC device for O_2_ evolution for the first time. This approach achieved a faradaic efficiency (FE) of 30% at an applied bias of 1.24 V *vs*. RHE, with a water oxidation potential matching the theoretical thermodynamic value. Moreover, their covalent attachment afforded fivefold higher photocurrent than that of laccase physically adsorbed controls, strongly supporting the notion that covalent-bonding is a viable strategy to promote electron transfer. Similarly, Efrati et al.^35^ utilized pyrroloquinoline quinone, an electron-accepting linker, to covalently anchor the PSI light-harvesting unit to ITO substrate through EDC chemistry. When further linked with glucose oxidase, an efficient electron flow wiring from the substrate to enzyme was evidenced by an obvious glucose concentration-dependent photocurrent response of the bio-hybrid PEC system. While covalent bonding can significantly enhance the interfacial mechanical stability of biocatalyst–substrate assemblies as compared to electrostatic interactions, more convenient bonding strategies remain under explored.

Interface layer modification: Modifying the substrate/biocatalyst interface can establish a more biocompatible local environment that protects biocatalysts from denaturation while enhancing their adhesion to the substrate. For semiconductor substrates, the interfacial layer can both enhance charge separation through composing built-in field, and protect the substrate surface against solvent-induced degradation^12,15,17^. Lee et al.^12^ coated a planar-TiO_2_ layer above p-Si photocathode, which further immobilized H_2_ase for H_2_ production. The TiO_2_ interface layer effectively protected the Si electrode surface from being oxidized in the aqueous environment, preventing the formation of an insulating SiO_2_ capping layer. Compared to the bare Si photocathode counterpart, TiO_2_-coated p-Si exhibited a nearly fivefold increase in photocurrent. Impressively, Kim et al.^20^ constructed a bias-free bio-hybrid PEC device by depositing a TiO_2_ modification layer onto nanowire-structured Si, followed by immobilization of *Sporomusa ovata*, a CO_2_-fixing bacterium, and coupling the system with anodic glycerol oxidation in a flow-cell configuration. This design achieved a bias-free photocurrent of approximately 1.2 mA cm^−2^, with FE exceeding 80% for both cathodic and anodic products. In a related study, we employed Cu_2_O, a promising p-type semiconductor with favorable band alignment and a theoretical photocurrent exceeding 14 mA cm^−2^, as the photocathode substrate^15^. To mitigate the inherent instability of Cu_2_O in aqueous conditions during operation, a ZnO interfacial layer was deposited via spin-coating. We found that the introduced ZnO layer not only stabilized the substrate but also introduced a Cu_2_O/ZnO p-n junction to promote charge separation. Following H_2_ase immobilization, the system achieved a photocurrent of 0.8 mA cm^−^^2^ at 0.15 V *vs*. RHE.

Besides inorganic interfacial layers, the organic polymer, e.g., polydopamine (PDA), has also been successfully utilized to establish a robust interface between semiconductor substrates and bacteria (*A. vinelandii*), as exemplified in the first bio-hybrid PEC for N_2_ fixation^17^. The authors observed a correlation between bacterial loading and PDA concentrations on the NiO substrate, revealing an enhanced bio-adhesive capability with higher PDA concentrations. Furthermore, they found that the bio-hybrid PEC device displayed significantly reduced charge transport impedance after the incorporation of a PDA layer. Consequently, compared to the bare NiO substrate, the PDA-modified NiO substrate achieved a nearly tenfold increase in photocurrent, with a champion NH_3_ production yield of 4.14 μmol h^−1^ cm^−2^. Also, the device showed no significant performance decline after 72 h of operation, highlighting its, in the context of biohybrid PEC devices, excellent durability.

### Mediated electron transfer

Direct electron transfer in bio-hybrid PEC systems critically requires precise orientation and intimate contact of the biocatalyst with the electrode substrate. However, achieving such optimal interfacing during device fabrication remains challenging. Introducing an electron mediator can overcome these limitations by shuttling electrons between the biocatalyst’s active site and the substrate, even when the bio-catalyst is sub-optimally oriented or in insufficient contact.

Protein mediator: Among mediators, cyt c, a heme protein redox mediator, provides several advantages: (i) High biocompatibility permit its seamless integration into diverse bio-hybrid architectures, including enzyme-, photosystem-, and whole cell-based systems, (ii) Owing to the intrinsic affinity to biocatalytic surfaces, it can be incorporated via simple solution-phase incubation, (iii) Relatively high thermodynamic stability beyond physiological conditions ensures a sustained mediator functionality, thereby enhancing operational durability^43,45,47,60–62^. Morlock et al.^45^ developed a series of PSI-immobilized electrodes, using cyt c as the mediator to drive O_2_ reduction. In the case of a 3D reduced graphene oxide as the electrode substrate, they observed increased photocurrent by more than tenfold and reduced overpotential by 100 mV after the addition of cyt c. Their further kinetic analyses found that electrodes incorporating cyt c exhibited a much more rapid photocurrent response than those lacking the redox protein. However, the mediator transport rate of cyt c has been recognized as a kinetic bottleneck that limits the overall efficiency in bio-hybrid PEC devices. To probe this limitation, Moort et al.^61^ conducted a systematic investigation into the charge transfer dynamics at both the “biocatalyst/cyt c” and “cyt c/substrate” interfaces. Their evaluation of photocurrent responses under varied conditions revealed that the dissociation rate of cyt c from the photosynthetic reaction center and its communication efficiency with the substrate are the rate-limiting steps in electron transfer processes, which need for further optimization.

Additionally, cyt c as redox mediator has been found to influence the operational and storage stability of the bio-hybrid PEC device. In a PEC device for quinone reduction, Friebe et al.^43^ revealed, aside from reactive oxygen species, the primary limitation to the device’s long-term performance was the mediator charge transfer efficiency between the substrate and the reaction center. In a month-long device stability test, a duration far exceeding the typical operational lifetimes of PSI (days) and PSII (minutes), the researchers observed recoverable photocurrents by periodically replenishing cyt c. Notably, after 10 days of operation, the photocurrent was approximately 60% higher in systems where cyt c was excluded from the working electrolyte. This finding indicates the critical role of optimizing the interfacial charge transfer via cyt c in ensuring long-term device stability and overall performance in bio-hybrid PEC systems. Despite these benefits, the relatively positive redox potential, approximately 0.2 to 0.35 V (*vs*. SHE) of cyt c limits its applications in high reduction potential required reactions. The relatively positive redox potential of cytc c originates from the π-acceptor properties of its axially coordinated methionine ligand, therefore selectively stabilizing the Fe²⁺ state over the Fe³⁺ state at the heme center^63,64^. If this can be tuned, it would significantly increase cyt c applicability in more varied catalysis systems.

Small molecule mediators: Small-molecule redox mediators are characterized by their rapid and reversible electron transfer kinetics, enabled fast diffusion and chemical tunability. Their redox potentials can be easily adjusted to match both biocatalyst and substrate, which makes them versatile and efficient electron shuttles in diverse bio-hybrid configurations. Commonly utilized small molecule mediators include hydroquinone derivatives^28,46,65^, metal complexes^66^, NADH^56,67^, viologen^68^, and gaseous species^29,69^ (e.g., H_2_, CO_2_). The adoption of whole-cell cyanobacteria eliminates the need for complex protein extraction and enhances biocatalyst stability through membrane protection; however, their insulating membranes significantly impede electron transfer efficiency. Chen et al.^28^ reported a 3D micropillar array ITO electrode via aerosol jet printing to effectively immobilize cyanobacterial biocatalysts, which then wired through the membrane-permeable mediator 2,6-dichloro-1,4-benzoquinone (DCBQ, with a redox potential of 0.32 V *vs*. SHE^70^). The bio-hybrid PEC device achieved a photocurrent of 245 μA cm^−2^ and an external quantum efficiency (EQE) of 29% under light intensities of 3 mW cm^−2^ and 1 mW cm^−^^2^, respectively. These values are approaching theoretical limits and represent a 110-fold enhancement over mediator-free controls, indicating DCBQ’s efficacy in bridging electron transfer between living cells and electrodes. Similarly, Tian et al.^46^ immobilized PSII on a polyethyleneimine-functionalized macro-porous carbon electrode for O_2_ evolution, and one order of magnitude augmented photocurrent was observed after incorporating DCBQ. They achieved an O_2_ evolution turnover number around 10200 over 10 h, and a stable photocurrent of 4.31 μA cm^−2^ under periodic irradiation over 5 days, indicating the important role of DCBQ in enhancing both catalytic activity and long-term durability.

Recently, Bouwens et al.^69^ developed an organic photovoltaic (OPV)-based photocathode onto which a series of enzymes were immobilized to catalyze cascade reactions for the production of chiral alcohols, utilizing CO_2_/HCOO^−^ as a sustainable redox mediator. In their device, CO_2_ was reduced to HCOO^−^ by a tungsten-containing FDH from *Nitratidesulfovibrio vulgaris*, which in turn drove NAD⁺-to-NADH conversion via an NAD⁺-dependent FDH from *Candida boidinii*. The resulting NADH was then used by alcohol dehydrogenase to reduce acetophenone to chiral 1-phenylethanol. Depending on the choice of alcohol dehydrogenase (ADH), either ADH_*S*_ or ADH_*R*_, the system enabled selective synthesis of (*S*)- or (*R*)−1-phenylethanol with high enantiomeric excess, reaching above 90%. Viologens are also commonly used redox mediators, with methyl viologen being particularly common for reduction processes, arguably due to its high reduction potential and commercial availability^68,71^. In this context, Gamache et al.^72,73^ has reported the effect of methyl viologen and structurally related diquat derivates as redox mediators on photocatalytic assemblies, revealing a large impact of the mediators on cell viability and catalytic performance.

Redox polymer mediator: Redox polymers as mediators in bio-hybrid PEC systems can both provide well-defined electron pathways to enhance electron transport efficiency, and serve as immobilization matrices to stabilize biocatalysts at the electrode interface. Moreover, the relatively weak diffusion properties of polymers also mitigate leaching issues, thus offering significant durability advantages over conventional small-molecule mediators. Polymer redox mediators can be classified into two categories: metal-centered redox polymers^22,23,37,39–42,44,74^ and pure organic polymers^16,25–27^. Os-complex redox polymers, firstly introduced by Badura, represents the most widely used metal-centered redox polymer^75^. For example, Zhao et al.^40^ employed an Os-complex redox polymer to wire PSI and PSII to the respective substrate, forming a bias-free water-splitting PEC device via a Z-scheme configuration by immobilizing PSI with H_2_ase. They found an accelerated electron transfer and reduced charge recombination after incorporating the Os-complex polymer, resulting in a substantial increase in photocurrent. Similarly, Sokol and colleagues developed a bio-hybrid TiO_2_ photoanode co-sensitized with an artificial dye and PSII for solar-driven water oxidation^22,23^. A Z-scheme electron transfer pathway was established between the dye and PSII via an Os-complex redox polymer, facilitating efficient O_2_ evolution at the photoanode. Simultaneously, CO_2_ reduction or H_2_ evolution occurred at the counter electrode, completing the bias-free solar-to-chemical conversion processes.

Beyond metal-centered redox polymers, Weliwatte et al.^26^ introduced an unbranched polydihydroxy aniline (PDHA), a pure organic redox polymer, for interfacing chloroplasts with conductive substrates. Unlike conventional branched redox polymers that rely on charge hopping among peripheral redox pendants and require favorable steric interactions, PDHA achieves high intrinsic electrical conductivity through charge transport by its well-oriented π-conjugated backbone. With the above design, they found a 4.2-fold increase in photocurrent compared to bare chloroplast-immobilized electrodes. While considerable progress has been made in developing bio-hybrid photoanodes, the advancement of photocathodes has been comparatively sluggish due to the limited availability of efficient photocathode semiconductors. To address this challenge, we developed a dye-sensitized NiO photocathode wired to H_2_ase through a viologen-based redox polymer (poly-V)^16^. Compared to control devices in absence of poly-V, more than twofold increase in the photocurrent and durability was observed after incorporating the redox polymer. The improvements are attributed to the fact that Poly-V can efficiently accept electrons from the reduced dye and transfer it to the H_2_ase, which significantly suppress charge recombination between the electron in the reduced dye or reduced H_2_ase and the injected hole in NiO. Moreover, it was found that the integration of the above system with a BiVO_4_-based photoanode to construct a bias-free tandem device for water-splitting yielded a solar-to-hydrogen conversion efficiency of 0.124%, while retaining over 75% of its initial photocurrent after 10 h of continuous operation.

The combination of two sorts of redox polymer, either metal-centered or purely organic, has also been explored as an effective strategy for charge mediation. For example, Tapia et al.^76^ employed a relatively positive Os-complex redox polymer to facilitate interfacial charge transfer between the electrode and PSI, generating reduced PSI. This reduced PSI then transfers electrons to H_2_ase via either a viologen-based or cobalt-complex redox polymer, achieving a positive HER onset potential of 0.38 V *vs*. SHE. While viologen-pendant polymers have been reported to scavenge O_2_ and protect H_2_ase from oxidative inactivation, the potentially more negative Co-complex polymer provides a higher overpotential for charge transfer. To fully harness the complementary properties of these redox materials, the development of multifunctional redox polymers is highly desirable.

### Future perspective

To enable large-scale applications of bio-hybrid PEC devices, efficient interface strategies have proven crucial, offering accelerated electron transfer kinetics, expanded catalytic versatility, and enhanced operational stability. While interface strategies are often classified by different electron transfer mechanisms, high-performance bio-hybrid PEC systems typically rely on the synergistic combination of multiple approaches rather than a single strategy. For instance, surface nano-structuring, a specific DET strategy, can increase the available surface area and provide more active sites for charge transfer, which actually is a critical requirement for both DET and MET. Importantly, achieving optimal device efficiency requires a co-optimized design of both the electrode substrate and the biocatalyst. As for the electrode substrate, semiconductor substrates serve dual roles as charge-collecting scaffold, also as light-harvesting and charge-separation units, addressing the inherent limitations of natural photosystems. Indeed, type I bio-hybrid PEC devices, which rely exclusively on natural light-harvesting complexes, often exhibit photocurrents several orders of magnitude lower than their type II counterparts that incorporate high-performance abiotic semiconductors (Table 1). Recent advances in type II systems, such as those employing heterojunction OPV materials, have demonstrated significantly enhanced photocurrents and catalytic activity, further highlighting their potential. On the biocatalyst side, subcellular components (e.g., isolated enzymes or organelles) often face challenges related to operational stability. In contrast, whole-cell biocatalysts offer several intrinsic advantages: ambient-condition stability, in situ regeneration of active components, and elimination of laborious protein extraction steps. Although the multilayered membranes in whole-cell systems enhance structural durability, they introduce additional charge transfer barriers that typically necessitate redox mediators. Further optimization of MET systems evidently depends on designing mediators that simultaneously offer efficient electron transfer, minimal leaching, and homogeneous wiring across large-area. Collectively, for industrial-scale applications, bio-hybrid systems that integrate abiotic semiconductors, whole-cell biocatalysts, and optimized redox mediators represent a highly attractive platform, especially in the production of high-value-added chemicals. Indeed, assembly of elaborate carbon-based products from simple starting materials (e.g., H_2_O and CO_2_) remain extremely challenging for purely abiotic PEC systems or individual enzyme bio-catalysts, but is relatively straightforward to achieve under whole-cell conditions through cascade reactions involving multiple enzymes.

**Table 1:** Photoelectrochemical catalysis parameters of the representative bio-hybrid PEC devices

Application	Light harvesting	Biocatalyst	Wiring method	Photocurrent	Stability test	Note
HER^40^	Au-PSI	H_2_ase	Poly-Vio Poly-Os (MET)	1.2 µA cm^−2^	9 min	Bias: 0.45 V *vs*. RHE Light: 160 mW cm^−2^ Tandem with PSII
HER^12^	p-Si	H_2_ase	Surface nanostructuring (DET)	1.4 mA cm^−2^	10 min	Bias: 0.1 V *vs*. RHE Light: 10 mW cm^−2^ FE: 35%
HER^16^	NiO-Dye	H_2_ase	Poly-Vio (MET)	141 ± 17 μA cm^−2^	5 h	Bias: 0 V *vs*. RHE Light: 50 mW cm^−2^ FE: 94% Tandem with BiVO_4_
CO_2_RR^19^ (formic acid)	ITO-PSI	FDH	Macroporous ITO (DET)	2.8 µA cm^−2^	22 h	Bias: 0.38 V *vs*. RHE Light: 100 mW cm^−2^ FE: 15%
CO_2_RR^18^ (formic acid)	Perovskite	FDH	Macroporous TiO_2_ (DET)	5 mA cm^−2^	10 h	Bias: 0.4 V *vs*. RHE Light: 100 mW cm^−2^ FE: 80 ± 10% Tandem with BiVO_4_
CO_2_RR^21^ (formic acid)	OPV	FDH	Macroporous TiO_2_ (DET)	3 mA cm^−2^	10 h	Bias: 0.6 V *vs*. RHE Light: 100 mW cm^−2^ FE: 90 ± 6% Tandem with Hematite
CO_2_RR^20^ (formic acid)	p-Si	*Sporomusa ovata*	Surface nanostructuring (DET)	1.1 mA cm^−2^	18 h	Bias: 0.15 V *vs*. RHE Light: 20 mW cm^−2^ Apply flow cell
ORR^47^	ITO-PSI-Dye	PS1	Cyt c (MET)	60.9 µA cm^−2^	2 h	Bias: 0.46 V *vs*. RHE Light: 100 mW cm^−2^
N_2_RR^17^	NiO	*A. vinelandii*	PDA (DET)	2.1 mA cm^−2^	72 h	Bias: −0.5 V *vs*. RHE Light: 100 mW cm^−2^ FE: 29%
Acetone reduction^69^	OPV	Multi-enzymes	Mesoporous TiO_2_ (DET)	1 mA cm^−2^	12 h	Bias: 0.8 V *vs*. RHE Light: 100 mW cm^−2^ FE: 10%
OER^22^	TiO_2_-Dye-PSII	PSII	Poly-Os (MET)	150 µA cm^−2^	1 h	Bias: 0.39 V *vs*. RHE Light: 100 mW cm^−2^ Tandem to TiO_2_-FDH
OER^28^	ITO-Cyanobacterium	Cyanobacterium	DCBQ (MET)	245 µA cm^−2^	2 min	Bias: 1 V *vs*. RHE Light: 3 mW cm^−2^ EQE: 29%
OER^34^	In_2_S_3_	Laccase	Covalent bond linking (DET)	40 µA cm^−2^	6 min	Bias: 1.4 V *vs*. RHE Light: 250 mW cm^−2^ FE: 45 ± 5%
Gluconic acid^35^	ITO-PSI	Glucose oxidase	Macroporous TiO_2_ (DET)	2 µA cm^−2^	70 s	Bias: 0 V *vs*. Ag QRE Light: 400 mW cm^−2^
Gluconolactone^37^	PbS	Glucose oxidase	Poly-Os (MET)	207 µA cm^−2^	30 min	Bias: 0.11 V *vs*. RHE Light: 100 mW cm^−2^

*HER* H_2_ evolution reaction, *CO*_2_*RR* CO_2_ reduction reaction, *ORR* O_2_ reduction reaction, *N*_2_*RR* N_2_ reduction reaction, *OER* O_2_ evolution reaction, *PS-I* photosystem I, *RHE* reversible hydrogen electrode, *p-Si* p-type silicon, *DET* direct electron transfer, *MET* mediated electron transfer, *ITO* indium tin oxide, *OPV* organic photovoltaic, *QRE* quasi-reversible electrode, *Bias* bias-voltage, *FE* faradaic efficiency.

**Fig. 3:**
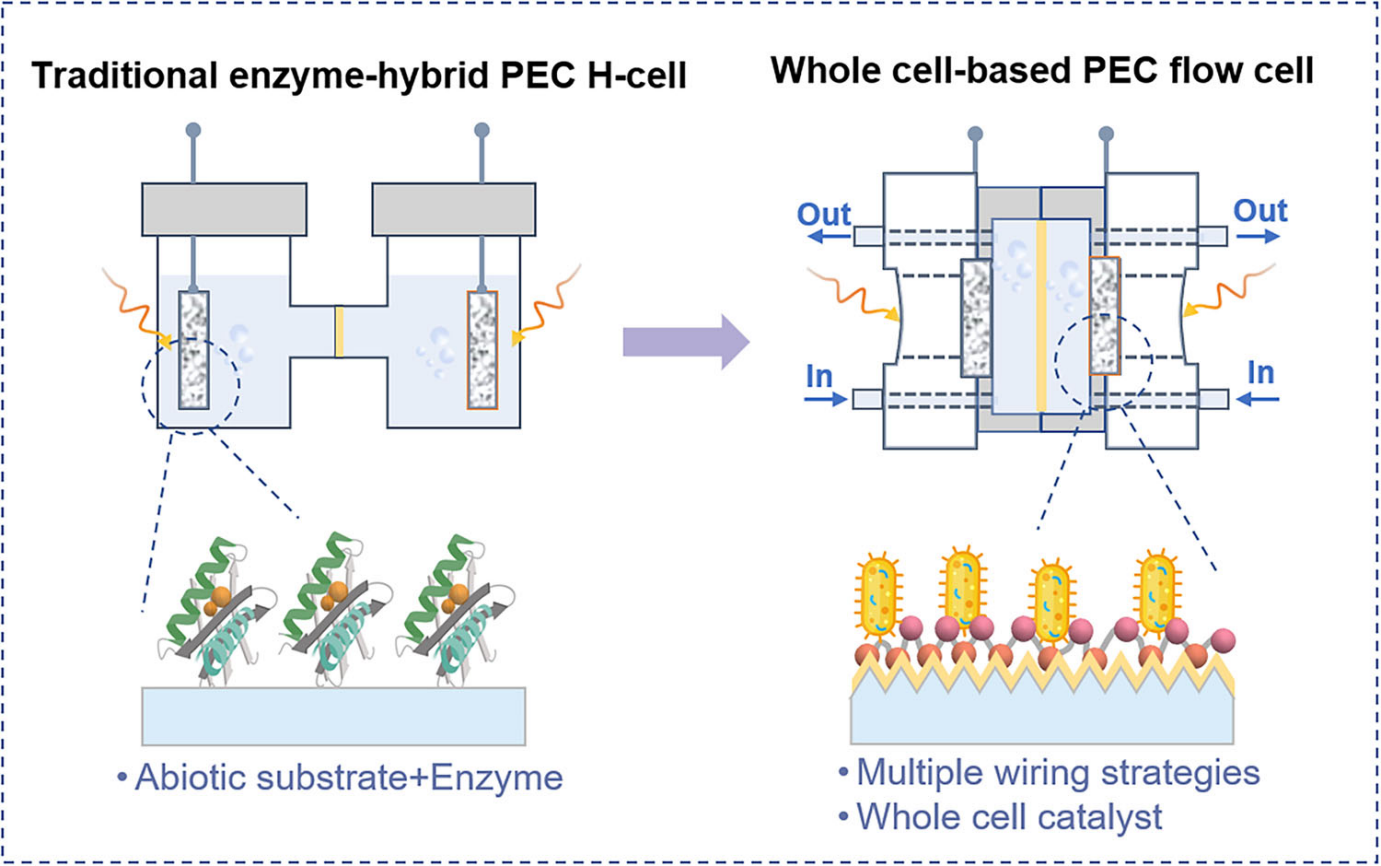
The developing trend of bio-hybrid PEC devices proposes a transition from conventional H-cell assemblies to bias-free tandem flow-cell architectures. Device structure of the H-cell with enzyme as the bio-catalyst and flow-cell with whole cell microbials as the bio-catalyst.

Using bio-hybrid PEC device for elaborate carbon-based compounds (C_x_H_y_O_z_) via the reduction of CO_2_ and H_2_O usually happens at the photocathode (H_2_O + CO_2_ + ne^−^ → C_x_H_y_O_z_). Although the reduction products are often the focus when discussing system output, their formation requires coupled oxidation reactions at the photoanode to supply the necessary electrons and protons. Thus, constructing bio-hybrid PEC devices into bias-free tandem configurations, driven solely by solar energy, has emerged as a key point in the research. To date, the OER has been the most widely coupling photoanodic process. Yet, its inherently sluggish kinetics severely constrain the overall efficiency of such tandem architectures. Substituting OER with alternative oxidation pathways, particularly kinetically favorable chemical valorization reactions, such as alcohol oxidation, offers a viable solution to overcome this kinetic challenge. Thus, although H_2_O is arguably the environmentally most appealing electron source, alternative anode substrates focusing on upgrading waste substrates into value-added products can improve the economic outlook of the system while still retaining many of the positive environmental aspects^20^. Moreover, replacing conventional H-cell assemblies with flow-cell reactors can substantially improve electrolyte micro-environment, mass and thermal management, and operational continuity, while simplify scaling-up production (Fig. 3). However, the lack of standardized catalytic testing protocols impedes direct comparison of the performance across different PEC devices, necessitating the establishment of unified benchmarking criteria. Specifically, key performance metrics should include photocatalytic activity measured under defined light intensities (e.g., 10 mW cm^−2^ and 100 mW cm^−2^ using a calibrated solar simulator), applied bias conditions (e.g., 0 V *vs*. RHE for hydrogen evolution and 1.23 V *vs*. RHE for oxygen evolution), FE over fixed time intervals (e.g., every 1 h), solar-to-hydrogen efficiency (ηₛₜₕ) for water-splitting in bias-free systems, and applied bias photon-to-current efficiency under externally biased conditions. Furthermore, relying solely on photocurrent for comparison can be misleading when assessing photoelectrochemical catalytic performance across different reactions, as this approach often overlooks variations in activation energy requirements. According to the Arrhenius Eq. (1), reaction kinetics decrease exponentially with increasing activation energy, making it a critical parameter for meaningful comparisons.

1 $$k=A{e}^{\frac{-\Delta {E}_{a}}{{RT}}}$$

It should also be noted that the activation barrier for a given pathway is not fixed but can vary significantly depending on the catalyst’s intrinsic properties. Adopting standardized evaluation protocols would greatly benefit the community as we advance towards scalable bias-free tandem flow-cell architectures, which integrates engineered whole-cell biocatalysts with high-performance semiconductors and their efficient interfacial wirings.
